# Heat shock proteins 70 and 90 from *Clonorchis sinensis* induce Th1 response and stimulate antibody production

**DOI:** 10.1186/s13071-017-2026-7

**Published:** 2017-03-01

**Authors:** Eun Joo Chung, Young-Il Jeong, Myoung-Ro Lee, Yu Jung Kim, Sang-Eun Lee, Shin-Hyeong Cho, Won-Ja Lee, Mi-Yeoun Park, Jung-Won Ju

**Affiliations:** Division of Malaria and Parasitic Diseases, Center for Immunology and Pathology, National Research Institute of Health, Korea Centers for Disease Control & Prevention, Osong, 28159 Republic of Korea

**Keywords:** *Clonorchis sinensis*, Heat shock protein, Immunogenicity, Peptide, Adjuvant

## Abstract

**Background:**

Heat shock proteins (HSPs) are found in all prokaryotes and most compartments of eukaryotic cells. Members of the HSP family mediate immune responses to tissue damage or cellular stress. However, little is known about the immune response induced by the oriental liver fluke, *Clonorchis sinensis*, even though this organism is carcinogenic to humans. We address this issue in the present study in mouse bone marrow dendritic cells (mBMDCs), using recombinant HSP70 and 90 from *C. sinensis* (rCsHSP70 and rCsHSP90).

**Methods:**

rCsHSP70 and rCsHSP90 were produced in an *E. coli* system. Purified recombinant proteins were treated in BMDCs isolated from C57BL/6 mice. T cells were isolated from Balb/c mice and co-cultured with activated mBMDCs. Expression of surface molecules was measured by flow cytometry and cytokine secretion was quantified using ELISA. C57BL/6 mice were divided into four groups, including peptide alone, peptide/Freund’s adjuvant, peptide/CsHSP70, peptide/CsHSP90, and were immunized intraperitoneally three times. Two weeks after final immunization, antibodies against peptide were measured using ELISA.

**Results:**

Both proteins induced a dose-dependent upregulation in major histocompatibility complex and co-stimulatory molecule expression and increased secretion of pro-inflammatory cytokines including interleukin (IL)-1β, -6, and -12p70 and tumor necrosis factor-α in mBMDCs. Furthermore, when allogenic T cells were incubated with mBMDCs activated by rCsHSP70 and rCsHSP90, the helper T cell (Th)1 cytokine interferon-γ was up-regulated whereas the level of the Th2 cytokine IL-4 was unchanged. These results indicate that rCsHSPs predominantly induce a Th1 response. Over and above these results, we also demonstrated that the production of peptide-specific antibodies can be activated after immunization via in vitro peptide binding with rCsHSP70 or rCsHSP90.

**Conclusion:**

This study showed for the first time that the HSP or HSP/peptide complexes of *C. sinensis* could be considered as a more effective vaccine against *C. sinensis* infection as results of the activator of host immune response as well as the adjuvant for antigenic peptide conjugate to induce peptide-specific antibody response in mice.

## Background

*Clonorchis sinensis* is a fish-borne parasitic trematode widely distributed in Korea, China, Taiwan, Vietnam, and Russia that is the causative agent of human clonorchiasis [1], which mainly occurs as a result of eating raw or undercooked freshwater fish infested with the metacercariae of *C. sinensis* [2–4]. Eating uncooked fish is also an important risk factor for intrahepatic CCA caused by clonorchiasis [5]. In 2009, *C. sinensis* was classified as a Group 1 carcinogen in humans by the International Agency for Research on Cancer [6]. The most common cause of death by clonorchiasis is cholangiocarcinoma (CCA) [7], a cancer of the bile ducts that is associated with chronic and severe liver fluke infection [1]; continuous stimulation of the bile duct by this organism induces pathological changes such as biliary mucosal hyperplasia, bile duct enlargement, periductal fibrosis, mechanical obstruction, inflammation, epithelial adenomatous hyperplasia, and biliary cirrhosis [1, 2, 8]. The association between liver flukes and CCA has also been demonstrated animal models [9]. Advanced CCA typically has poor prognosis, with a median survival time of < 24 months [5, 10]; around 5,000 cases of CCA attributed to *C. sinensis* infection are reported worldwide each year [11]. A survey in Korea showed that the ratio of patients with CCA differed significantly between those with and without clonorchiasis [12]. As such, considerable effort has been focused on the development of a vaccine against *C. sinensis*. One study reported the protective effect of Cs14-3-3 epsilon protein [13] while another showed that CsTP22.3 expressed by *Bacillus subtilis* spores was immunogenic and could be orally administered to provide protection against *C. sinensis* infection [14].

Heat shock proteins (HSPs) constitute a highly conserved family in most organisms that provide cellular protection under stressful conditions including heat, oxidative stress, glucose starvation, irradiation and viral infection [15]. HSP70 and HSP90 provide a link between innate and adaptive immune responses via activation of lymphocytes and antigen-presenting cells (APCs) such as dendritic cells (DCs) [15, 16]. HSPs purified from bacterial and mammalian sources are potent stimulators of the innate immune response [16], which includes production of pro-inflammatory cytokines such as tumor necrosis factor (TNF)-α and interleukin (IL)-1β, -6, and -12 by macrophages [17] and DCs [18, 19] and the upregulation of surface markers such as cluster of differentiation (CD)40, CD80, and CD86 in DCs [20]. HSPs also induce antigen-dependent T cell activation as well as interferon (IFN)-γ secretion [21, 22], and are associated with peptides that are presented by major histocompatibility complex (MHC), MHC II and MHC I on APCs [23–25]. Antigenic peptide/HSP70 complexes activate DCs for cytokine release and prime cytotoxic T lymphocyte (CTL) responses [18, 26]. Mice immunized with HSP/CTL epitope peptide complexes produced CTLs and showed a peptide-specific antibody response [27]. Various studies have shown that immunization with parasite HSPs induce cellular and humoral immune responses and protect against infection by the parasite [20, 28–31]. These findings suggest that pathogen HSPs are a candidate vaccine against infectious diseases. However, no studies have been done on host immune responses to HSPs in trematodes, including *C. sinensis* despite its risk to humans.

We addressed this in the present study by investigating in vitro immune responses to *C. sinensis* HSP70 and HSP90. Their adjuvant effect was confirmed by analyzing anti-peptide antibody production after immunization of peptide bound to CsHSP70 and CsHSP90 in a murine model.

## Methods

### Recombinant *C. sinensis* (rCsHSP) expression and purification

cDNA clones encoding CsHSP70 and CsHSP90 were obtained from the adult *C. sinensis* cDNA library. The open reading frames of the two proteins were PCR-amplified using the following forward and reverse primer sets: CsHSP70, 5'-GAG CGA TCT CAT GTC GAA GGT CAT GCT-3' and 5'-CGC GTC GAC CTA TTG TTT CTG CTG AG-3'; and CsHSP90, 5'-GCA ATT CCA TAT GTC TTG CGA ACC GAT GGC-3' and 5'-CCC AAG CTT ATC GAC TTC TTC CAT TCC AGC-3'. The PCR products were cloned into the pET28a and pET21a vectors (Novagen, Madison, WI, USA), respectively. Recombinant plasmids were transformed into *Escherichia coli* BL21 (DE3)-RIPL cells, which were grown in Luria-Bertani medium containing ampicillin or kanamycin. Isopropyl-B-d-thiogalactopyranoside was added to the culture to a final concentration of 0.5 mM and cells were incubated for 20 h at 16 °C, then harvested by centrifugation and resuspended in lysis buffer [50 mM NaH_2_PO_4_, 300 mM NaCl, and 5 mM imidazole (pH 8.0)]. The cell suspension was sonicated on ice, and supernatants were collected after centrifugation. Recombinant proteins were purified using Ni-nitrilotriacetic acid agarose (Qiagen, Valencia, CA, USA) under native conditions. Briefly, supernatants were loaded in a column pre-equilibrated with lysis buffer. The column was washed with five bed volumes of wash buffer [50 mM NaH_2_PO_4_, 300 mM NaCl, and 20 mM imidazole (pH 8.0)] and then eluted with elution buffer [50 mM NaH_2_PO_4_, 300 mM NaCl, 250 mM imidazole (pH 8.0)]. The eluted protein fraction was concentrated using Amicon Ultra-4 centrifugal filter devices (Millipore, Billerica, MA, USA) and protein concentration was estimated by the Bradford method using bovine serum albumin as a standard. The purity of eluted proteins was evaluated by sodium dodecyl sulfate polyacrylamide gel electrophoresis (SDS-PAGE) and sequences were confirmed by liquid chromatography–tandem mass spectrometry (LC-MS/MS). Recombinant proteins were dialyzed against Dulbecco’s phosphate buffered saline (DPBS; pH 7.4) for mouse immunization or treatment of mouse bone marrow dendritic cells (mBMDCs).

Before experiments, endotoxin contaminants of rCsHSP70 and rCsHSP90 were removed using Detoxi-Gel endotoxin removal columns (Pierce, Rockford, IL, USA). The endotoxin level in recombinant proteins was determined with the *Limulus amoebocyte* lysate (LAL) Chromogenic Endotoxin Quantitation kit (Pierce) and was found to be negligible (<1 EU/ml). Additionally, rCsHSPs were pre-incubated with polymyxin B (Sigma-Aldrich, St. Louis, MO, USA) before being used for mBMDC treatment.

### mBMDC preparation

Femurs and tibiae were removed from C57BL/6 mice and washed with DPBS. Both ends of isolated bones were cut with scissors and the bone marrow was flushed out with ice-cold DPBS using a syringe. After one wash with DPBS, erythrocytes were lysed by treatment with red blood cell lysis buffer (Sigma-Aldrich). mBMDCs were obtained by culturing the cells (2 × 10^6^ cells/ml) in the presence of 20 ng/ml recombinant granulocyte–macrophage colony-stimulating factor (GM-CSF; Peprotech, Rocky Hill, NJ, USA) for 8 days in RF10 medium (Roswell Park Memorial Institute (RPMI)-1640 medium (Gibco, Carlsbad, CA, USA) supplemented with 10% fetal bovine serum (FBS; Gibco), 1% penicillin-streptomycin, and l-glutamine). The medium was replaced every 3 days.

### Analysis of DC-secreted cytokines and DC surface markers

After 8 days, cells were harvested and resuspended in RF10 medium containing GM-CSF. Cells were then seeded in a 6-well plate at a density of 2 × 10^6^ cells/well. rCsHSPs or proline-rich (ProR) peptide were mixed with 50 μg/ml polymyxin B and incubated at 4 °C. After 1 h, immature DCs were stimulated with rCsHSP70, rCsHSP90, ProR peptide, or 0.1 μg/ml lipopolysaccharide (LPS; Sigma-Aldrich) as a positive control for 18 h in humidified 5% CO_2_ incubator at 37 °C. After treatment, cells were harvested and centrifuged at 2000× *g* for 5 min. The supernatant was collected and stored at −20 °C until use. Cells were then labeled with fluorescein isothiocyanate (FITC)-conjugated anti-mouse CD11c antibody; phycoerythrin (PE)-conjugated anti-mouse CD40, CD80, or CD86 antibody; or allophycocyanin (APC)-conjugated MHC Class II (I-A/I-E) or MHC Class I (H-2 kb) antibody (all from eBioscience, San Diego, CA, USA) for 30 min at 4 °C. After 30 min of labeling, cells were washed with DPBS and resuspended in 4% paraformaldehyde for analysis with a FACSVerse flow cytometer (BD Biosciences, Franklin Lakes, NJ, USA); data were analyzed using FlowJo software (Ashland, OR, USA). Levels of interleukin (IL)-6, -1β, -12p70, and -10 and TNF-α in culture supernatants were quantified with an enzyme-linked immunosorbent assay (ELISA) kit (R&D Systems, Minneapolis, MN, USA), according to the manufacturer’s instructions.

### Measurement of intracellular cytokine levels in DCs

Intracellular cytokines were detected using the Cytofix/Cytoperm kit (BD Biosciences), according to the manufacturer’s instructions. Briefly, DCs (1 × 10^6^ cells/ml) treated with rCsHSPs were harvested and centrifuged at 1500× *rpm* for 5 min. The supernatant was removed and the pellet was washed with staining buffer (2.5% FBS in DPBS), and 1 μg/ml of FITC-conjugated anti-mouse CD11c IgG (eBioscience) was added followed by incubation at 4 °C for 30 min. The pellets were washed twice with staining buffer and cells were collected by centrifugation. The pellets were resuspended and incubated in 250 μl of Cytofix/Cytoperm solution (BD Biosciences) for 20 min at 4 °C, then washed twice in 1× Perm/Wash solution (BD Biosciences). To label intracellular cytokines, cells were incubated in 1 μg/ml PE-conjugated anti-mouse IL-10 or-12 or IFN-γ IgG solution (eBiosciences) at 4 °C for 30 min in the dark, then washed twice in 1× Perm/Wash solution and fixed with 4% paraformaldehyde at 4 °C. The fluorescence intensity of intracellular cytokines was measured by flow cytometry.

### Endocytosis test

To analyze the endocytic capacity of DCs, 1 × 10^6^ cells were incubated in the presence or absence of rCsHSPs for 20 h. The cells were harvested and washed twice with DPBS, and 1 × 10^6^ cells were incubated at 37 °C or 4 °C for an additional 30 min with 1 mg/ml FITC-dextran (40,000 MW anionic, lysine-fixable; Molecular Probes, Eugene, OR, USA). After incubation, cells were washed twice with ice-cold staining buffer (0.1% FBS and 0.01% NaN_3_ in PBS), and 1 μg/ml PE-conjugated anti-mouse CD11c IgG (eBioscience) was added followed by incubation at 4 °C for 30 min. After two washes, labeled DCs were analyzed by flow cytometry.

### Allogenic mixed lymphocyte reaction (MLR)

T cells were prepared from splenocytes obtained by grinding spleens of 8-week-old BALB/c mice. CD3^+^ T cells were purified via negative selection using magnetic-activated cell sorting (MACS) columns (Pan T Cell Isolation Kit II; Miltenyi Biotec, San Diego, CA, USA). Isolated T cells were stained with carboxyfluorescein succinimidyl ester (CFSE) using the Cell Trace CFSE Cell Proliferation kit (Molecular Probes). BMDCs (5 × 10^4^ cells/well) were stimulated with 10 μg/ml rCsHSPs for 24 h, washed twice, and then co-cultured with CFSE-labeled T cells (5 × 10^5^ cells/well). Mixed cells were added to a 96-well round-bottom plate and cultured in humidified 5% CO_2_ incubator at 37 °C. Cells were harvested after 4, 5, and 6 days and supernatants were collected and stored at −20 °C until analysis. Harvested cells were washed twice and then stained with 1 μg/ml PE-conjugated anti-mouse CD3e antibody (eBioscience) at 4 °C for 30 min. After two washes with DPBS, the cells were resuspended in 4% paraformaldehyde and fluorescence intensity was measured by flow cytometry. IL-2 and -4 and IFN-γ concentrations in the cell culture supernatant were measured with ELISA kits (R&D Systems), according to the manufacturer’s instructions.

### rCsHSPs immunization and measurement of cytokines secreted from splenocytes

Groups of 5 C57BL/6 mice were immunized intraperitoneally with 50 μg of rCsHSP70 or rCsHSP90 emulsified in complete Freund’s adjuvant (CFA). Subsequent booster doses were given on 14th and 28th day with 50 μg of rCsHSP70 or rCsHSP90 emulsified in incomplete Freund’s adjuvant (IFA). The control mice received an equal volume of PBS mixed with adjuvant. After the 7th day of the last immunization, the splenocytes were isolated from mice and cultured (1 × 10^5^ cells/well) either in the absence or presence of 5 μg/ml of rCsHSPs for 72 h. The levels of IL-2, -4 and IFN-γ in culture supernatants were determined using ELISA kits (R&D system), according to the manufacturer’s instructions.

### rCsHSPs/CsProR peptide complex formation

CsProR peptide (> 95% purity; DAPVPKSGGPDAPVPKSGGPDAPVPKSGG) was synthesized by Peptron (Daejeon, Korea). The ProR sequence was derived from the proline-rich antigen of *C. sinensis* (GenBank accession no. AAK35165). To prepare rCsHSPs/CsProR peptide complexes, CsHSP70 or CsHSP90 was mixed with CsProR peptide for 1 h in binding buffer [1 mM ADP and 1 mM MgCl_2_ in DPBS (pH 7.4)] at 37 °C with shaking. After 1 h of incubation, 50 μg/ml polymyxin B was added, and complexes were incubated at 4 °C for 1 h, then immediately transferred to ice until use.

### Immunization with CsHSPs/ProR peptide complexes

C57BL/6 mice were divided into four groups of five animals each as follows: group 1 was immunized with ProR peptide alone; group 2 was immunized with ProR peptide combined with Freund’s adjuvant (FA); group three was immunized with CsHSP70/ProR peptide complexes; and group four was immunized with CsHSP90/ProR peptide complexes. Mice were intraperitoneally injected three times at 2-week intervals with complete FA for the first injection and incomplete FA for the first and second boosts. After final immunization, whole blood was collected from each mouse by cardiac puncture and centrifuged at 2000× *rpm* and 4 °C for 10 min. Obtained serum samples were stored at -20 °C until analysis.

### Analysis of T cell proliferation

Splenocytes were prepared by grinding the spleens of five immunized mice from each group. T cells were purified from splenocytes via negative selection using MACS columns. Prepared T cells were cultured in a 96-well plate at a density of 1 × 10^5^ cells/well in a final volume of 100 μl/well after resuspension in cell culture medium (RPMI 1640 with 10% FBS and 1% antibiotics). The plate was incubated in the absence or presence of ProR peptide (10 μg/ml) at 37 °C in a 5% CO_2_ incubator for 72 h before 10 μl/well WST-1/electro coupling solution (BioVision, Milpitas, CA, USA) was added to each well. The cells were incubated under standard culture conditions for 4 h, and sample absorbance was measured using a microplate reader at 450 nm (reference wavelength: 620 nm).

### Determination of antibody titer

Humoral immune responses elicited by ProR peptide/rCsHSPs complexes were analyzed by detecting the production of specific antibodies against the peptide in CsProR peptide-coated ELISA plates. Briefly, streptavidin-coated 96-well plates (Thermo Fisher Scientific, Waltham, MA, USA) were incubated with 5 μg/ml biotin-conjugated CsProR peptide for 18 h at 4 °C. All subsequent steps were carried out at room temperature. After three washes with PBS containing 0.1% Triton X-100 (PBST), plates were blocked with blocking buffer (5% skim milk in PBST) for 2 h, washed, and incubated in immunized mouse serum diluted in blocking buffer for 2 h. After washing, 100 μl/well horseradish peroxidase-conjugated rabbit anti-mouse IgG (Sigma-Aldrich) diluted 1:10,000 in blocking buffer were added, followed by incubation for 2 h; 50 μl/well 3,3′,5,5′-tetramethylbenzidine/H_2_O_2_ substrate were added, followed by incubation for 30 min. The enzymatic reaction was terminated by adding 50 μl/well of 2 N H_2_SO_4_ to the wells and optical density was measured with a spectrophotometer at 450 nm (reference wavelength: 620 nm). For measuring IgG isotypes, detection of antibodies was determined using either horseradish peroxidase-conjugated goat anti-mouse IgG1 or IgG2a (BioRad, Hercules, CA, USA).

### Statistics analysis

Statistical analyses were performed using two-tailed *t-*tests or 2 way ANOVA run on GraphPad Prism version 5.01 software (GraphPad Software, San Diego, CA). Experimental data were presented as the standard error of the mean (SEM). Differences between groups were considered significant if *P-*value was less than 0.05.

## Results

### Purification of rCsHSP70 and rCsHSP90

**Fig. 1:**
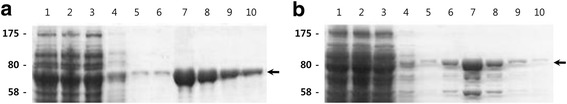
SDS-PAGE images of rCsHSPs expression. a rCsHSP70 and b rCsHSP90 were purified under native conditions and resolved by 10% SDS-PAGE. Molecular weight markers are indicated in *left*. Lane 1: total induced cell lysate; Lane 2: supernatant (sup); Lane 3: flow through; Lanes 4–5: wash; Lanes 6–10: eluted fraction. rCsHSPs are indicated by *arrows*

CsHSP70 and CsHSP90 are 656 and 714 amino acids, respectively, with predicted molecular weights of 75 and 83.3 kDa, respectively. The expression of these proteins was confirmed by the band size in the SDS-PAGE analysis (Fig. 1); the purified proteins had significant homology (*P* < 0.05) to the known sequences of CsHSP70 and CsHSP90, as determined by LC-MS/MS.

### rCsHSPs increase surface marker expression and cytokine production in mBMDCs

**Fig. 2:**
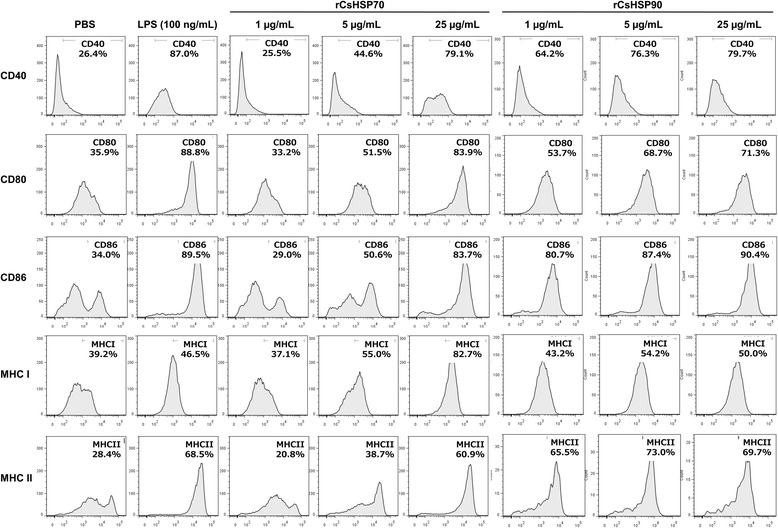
Increase in expression of BMDC surface markers by rCsHSPs. BMDCs from C57BL/6 mice were cultured for 7 days and then incubated for 22 h with rCsHSP70 or rCsHSP90. Cells were harvested and labeled with FITC-conjugated anti-mouse CD11c or PE-conjugated anti-mouse CD40, CD80, CD86, MHCI, or MHCII antibodies on ice for 30 min. Fluorescence intensity was measured by flow cytometry

rCsHSP70 and rCsHSP90 with endotoxins removed induced the expression of co-stimulatory molecules by mBMDCs in a dose-dependent manner (Fig. 2). rCsHSP induced mBMDC maturation, as demonstrated by the upregulation of the co-stimulatory molecules CD40, CD80, and CD86, whereas treatment with either protein dose-dependently increased MHC I and MHC II expression. Moreover, MHC I expression was higher than in the LPS-treated positive control group.

**Fig. 3:**
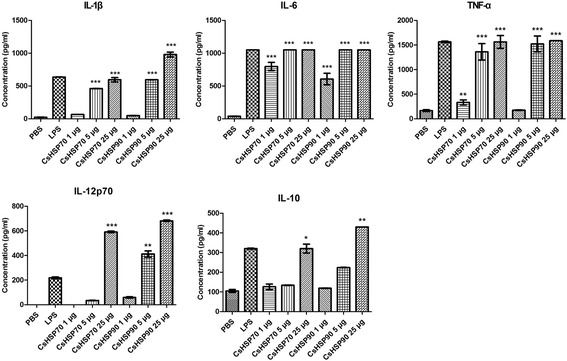
rCsHSPs dose-dependently induce cytokine secretion by BMDCs. BMDCs from C57BL/6 mice were cultured for 7 days and then incubated for 22 h with rCsHSP70 or rCsHSP90. Secreted cytokine levels in culture supernatants were measured by ELISA. The results are shown as the mean ± standard error (SEM). *P < 0.05, **P < 0.01, ***P < 0.001 compared to PBS-treated group

**Fig. 4:**
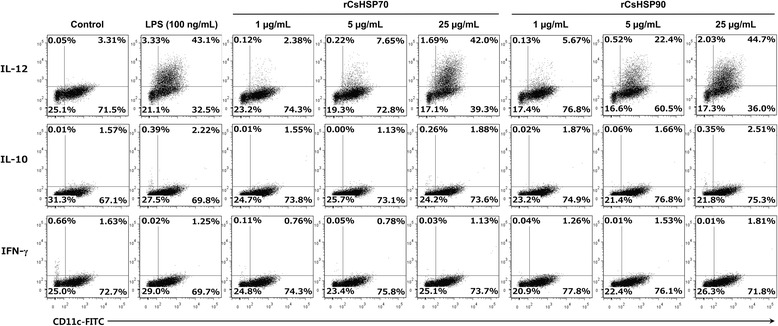
rCsHSPs increase production of intracellular IL-12 in CD11c^+^ mBMDCs. mBMDCs were stimulated with rCsHSP70 and rCsHSP90 for 18 h, then labeled with FITC-conjugated CD11c antibody before permeabilization. Intracellular cytokines were labeled with PE-conjugated antibodies against mouse IL-12, IL-10, and IFN-γ. The number of cytokine-producing cells was determined by flow cytometry

Cytokines secreted from activated DCs are classified as Th2-type cytokines (e.g., IL-10) or pro-inflammatory cytokines (e.g., IL-12p70, -6, and -1β and TNF-α) [18, 32]. We measured the concentration of these cytokines secreted from mBMDCs by ELISA in cell culture supernatants. There was a dose-dependent increase in the secreted levels of pro-inflammatory cytokines (IL-12p70, -6, and -1β and TNF-α) following treatment with rCsHSP70 (*t*_(2)_ = 12.25, *P* = 0.0066) or rCsHSP90 (*t*_(2)_ = 16.22, *P* = 0.0038). IL-10 level was also slightly increased by high concentrations of rCsHSP70 (*t*_(2)_ = 9.065, *P* = 0.012) or rCsHSP90 (*t*_(2)_ = 27.01, *P* = 0.0014) (Fig. 3). In addition, measurement of intracellular IL-10 and -12 production by flow cytometry revealed an increase in IL-12 level according to rCsHSP concentration, whereas IL-10 was not detected (Fig. 4).

### rCsHSP70 and rCsHSP90 are taken up as antigens by mBMDCs

**Fig. 5:**
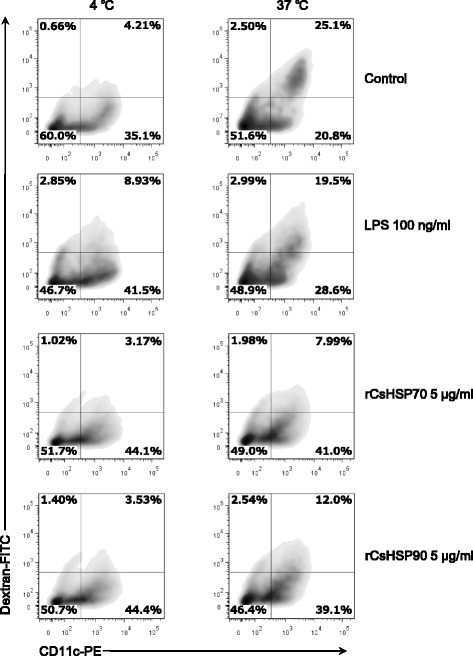
Reduced endocytic capacity of BMDCs by rCsHSP70 and rCsHSP90 treatment. mBMDCs treated with rCsHSPs or LPS were incubated with FITC-labeled dextran for 30 min at 4 °C or 37 °C. Endocytic capacity was analyzed by flow cytometry

DCs mature following antigen uptake and subsequently decrease their endocytic activity [33, 34]. We investigated whether rCsHSPs affect the endocytic capacity of mBMDCs treated with FITC-conjugated dextran and activated with rCsHSPs (Fig. 5). Immature mBMDCs captured a large amount of dextran-FITC at 37 °C relative to the non-specific control at 4 °C. This uptake was abrogated upon treatment with 5 μg/ml rCsHSPs to a level similar to that induced by the positive control LPS (0.1 μg/ml).

### mBMDCs activated by CsHSP70 and CsHSP90 induce T cell proliferation and differentiation

**Fig. 6:**
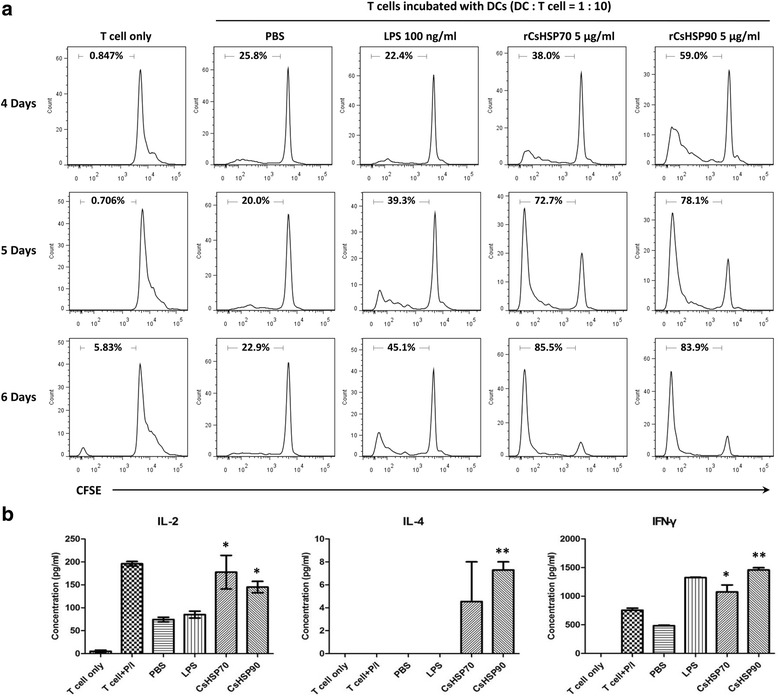
mBMDCs matured by stimulation with rCsHSPs show increased T cell proliferation and T cell secretion of IL-2 and IFN-γ. mBMDCs were incubated overnight with rCsHSP70, rCsHSP90, or LPS as indicated. mBMDCs were then washed and co-cultured with CFSE-labeled T cells. **a** T cell proliferation was evaluated after 4, 5 and 6 days by flow cytometry. X- and y-axes indicates fluorescence intensity for CFSE and number of cells, respectively. **b** IL-2 secretion was quantified after 16 h; IL-4 and IFN-γ levels were measured after 5 days by ELISA. The results are shown as the mean ± standard error (SEM) and are representative of three independent experiments. *P < 0.05, **P < 0.01 compared to PBS-treated group

To further investigate the cellular response elicited by rCsHSPs, we evaluated T lymphocyte proliferation and cytokine secretion in response to stimulation with 5 μg/ml rCsHSPs. mBMDCs activated with rCsHSPs were co-cultured with T cells isolated from splenocytes of allogenic mice; T cell activation was confirmed by MLR. The number of T cells stimulated with mature BMDCs increased in a time-dependent manner (Fig. 6a). In addition, IL-2 (CsHSP70; *t*_(2)_ = 5.322, *P* = 0.0335, CsHSP90; *t*_(2)_ = 5.257, *P* = 0.0343) and IFN-γ levels (CsHSP70: *t*_(2)_ = 4.85, *P* = 0.04; CsHSP90: *t*_(2)_ = 23.83, *P* = 0.0018) in T cells were increased relative to the PBS-treated group in response to rCsHSPs stimulation, although IL-4 level was slightly increased in case of rCsHSP90-treatment (*t*_(2)_ = 10.15, *P* = 0.0096) (Fig. 6b).

### rCsHSP70 and rCsHSP90 stimulate IFN-γ secretion from splenocytes of immunized mice

**Fig. 7:**
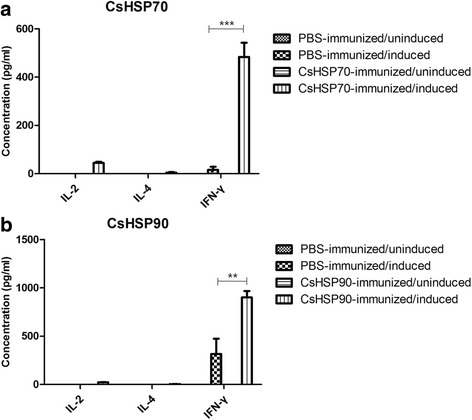
Cytokines secreted from splenocytes of PBS or rCsHSPs-immunized mice. Mice were immunized on day 0 with PBS or 100 μg rCsHSPs per mouse emulsified in complete Freund’s adjuvant followed by two booster injections with 100 μg rCsHSPs per mouse emulsified in incomplete Freund’s adjuvant on day 14th and 28th days. After the 7th day of the last immunization, the splenocytes were isolated from mice and were cultured either in the absence or in the presence of 5 μg/ml rCsHSP70 (**a**), rCsHSP90 (**b**) for 72 h. The results are shown as the mean ± standard error (SEM) and are representative of three independent experiments. **P < 0.01, ***P < 0.001

We also measured cytokines secreted from splenocytes after re-stimulation in rCsHSPs-immunized mice (Fig. 7). The levels of IFN-γ produced in the splenocytes in response to rCsHSP70 (*t*_(8)_ = 7.757, *P* < 0.0001) and rCsHSP90 (*t*_(8)_ = 3.398, *P* = 0.0094) stimulation were significantly higher in cells isolated from immunized mice as compared to PBS-immunized control mice. But IL-2 and IL-4 were not changed. This result was consistent with the results of in vitro experiments.

### rCsHSPs/ProR peptide complexes increase cytokine secretion from mBMDCs

**Fig. 8:**
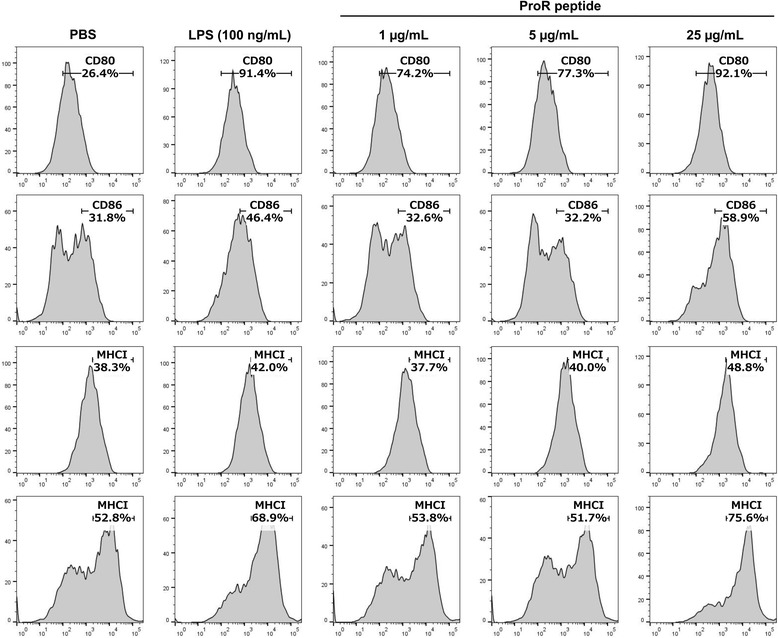
Surface markers expression in BMDCs treated with CsProR peptide. BMDCs were treated with 1, 5 and 25 μg/ml ProR peptide and then labeled with FITC-conjugated anti-mouse CD11c or PE-conjugated anti-mouse CD80, CD86, MHCI or MHCII antibodies on ice for 30 min. Fluorescence intensity was evaluated by flow cytometry

**Fig. 9:**
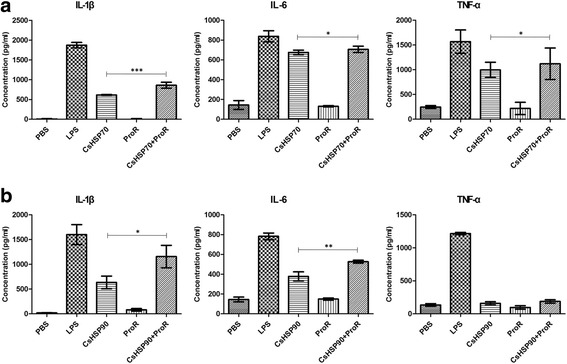
Cytokines secretion by BMDCs induced by CsHSP/ProR peptide complexes. rCsHSP70 or rCsHSP90 (1 μg/ml) was mixed with ProR peptide at a 200:1 peptide:protein molar ratio in binding buffer at 37 °C for 1 h. After polymyxin B treatment for 1 h, BMDCs were treated with rCsHSP70, rCsHSP90, CsProR peptide or CsHSP/ProR complexes. Levels of secreted cytokines were measured by ELISA. Panel **a** shows results by CsHSP70/CsProR and panel **b** shows results by CsHSP90/CsProR. The results are shown as the mean ± standard error (SEM). *P < 0.05, **P < 0.01, ***P < 0.001

HSPs act as adjuvants in antigen presentation. To verify whether CsHSPs serve this function, we examined mBMDC activation by CsHSP70 or CsHSP90 bound to an antigenic peptide that cannot be presented as an antigen, by itself. ProR peptide of *C. sinensis* is a strong antigenic peptide that we previously detected in the sera of clonorchiasis patients (data not shown); we therefore tested whether the ProR peptide alone or in a complex with rCsHSP70 or rCsHSP90 could induce cellular immunity. When mBMDCs were stimulated with ProR peptide alone, surface molecule expression increased only at high concentration (Fig. 8) and cytokine secretion were unaltered (Fig. 9). However, CsHSP70/ProR peptide complexes increased TNF-α (*t*_(2)_ = 4.501, *P* = 0.046) and IL-6 (*t*_(2)_ = 9.817, *P* = 0.0102) and -1β (*t*_(2)_ = 40.91, *P* = 0.0006) secretion from mBMDCs, and CsHSP90/ProR peptide complexes also stimulate IL-6 (*t*_(2)_ = 12.66, *P* = 0.0062) and -1β (*t*_(2)_ = 4.821, *P* = 0.0404) secretion by mBMDCs (Fig. 9), although surface marker expression was unaffected (data not shown).

### rCsHSPs stimulate CsProR peptide-specific antibody production

**Fig. 10:**
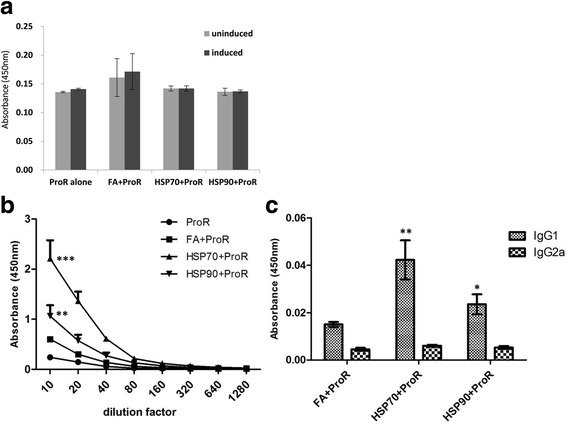
rCsHSPs stimulate the production of anti-CsProR peptide antibodies via peptide interaction. Freund’s adjuvant (FA) or rCsHSPs (30 μg/ml) was incubated with CsProR peptide (100 μg/ml) for 1 h at room temperature. C57BL/6 mice were immunized with peptide immersed in FA or rCsHSP/peptide complexes. **a** T cells and sera were obtained from immunized mice on day 7 after final immunization. T cells were treated with PBS or 10 μg/ml CsProR peptide for 72 h, and proliferation was then evaluated by WST-1 assay. Anti-CsProR antibodies (**b**) and their isotypes (**c**) detected in immunized mice serum by ELISA. The results are shown as the mean ± standard error (SEM). *P < 0.05, **P < 0.01, ***P < 0.001 compared to PBS-immunized mice group

We next examined whether CsHSP70 and CsHSP90 induce production of peptide-specific antibodies. To assess in vivo immune responses, ProR peptide was mixed with rCsHSPs or FA and used to immunize mice. T cells isolated from the splenocytes of immunized mice were re-stimulated with 10 μg/ml ProR peptide, and anti-ProR antibody levels were measured in mouse serum. The T cell proliferation test revealed that the number of T cells did not increase in mice immunized with the peptide mixed with rCsHSPs or FA (Fig. 10a). However, the production of antibodies against ProR peptide was enhanced in mice immunized with CsHSP70/ProR peptide (*F*_(7,7)_ = 86.69, *P* < 0.0001) or CsHSP90/ProR peptide (*F*_(7,7)_ = 18.51, *P* = 0.001) complexes (Fig. 10b), with a stronger effect observed for rCsHSP70 than for rCsHSP90. Moreover, to determine the type of immune response, the isotype IgG1 and IgG2a was determined using ELISA. The results revealed that IgG1 levels were higher than IgG2a levels in both rCsHSP70/ProR (*F*_(3,3)_ = 58.91, *P* = 0.0073) and rCsHSP90/ProR (*F*_(3,3)_ = 15.21, *P* = 0.05) immunized (Fig. 10c). This result indicates that rCsHSP/ProR complexes polarize the response towards the Th2 immune response in mice. Antibody isotyping results showed that rCsHSPs can induce Th2 immune response as well as Th1 response effectively.

## Discussion

HSP acts as a molecular chaperone that enhance host immune responses [15, 16], and represent dominant antigens in many infectious diseases. Given their importance in host-pathogen immune interactions, these proteins are considered as vaccine candidates [35]. The present study showed that two recombinant HSPs derived from *C. sinensis*, rCsHSP70 and rCsHSP90, can stimulate murine DCs and T cells, as evidenced by the increase in co-stimulatory molecule expression and cytokine secretion in the presence of these proteins. We also found that rCsHSPs can induce production of antigenic peptide-specific antibodies.

Recombinant HSPs are often contaminated with bacteria-derived endotoxins, which are responsible for most in vitro cytokine effects of HSPs [16, 36]. However, the immunogenicity of HSPs with low endotoxin levels in DCs has also been reported [18, 19]. Commonly used methods for eliminating endotoxin contamination in experiments using immune cells include endotoxin measurement using the LAL assay, and co-incubation of recombinant HSPs with the neutralizing agent polymyxin B [37]. In this study, we removed endotoxins from rCsHSPs with the LAL assay (endotoxin level < 1 EU/ml); as an added precaution, the proteins were pre-incubated with polymyxin B before they were used in experiments.

Pathogen-derived HSPs induce an innate immune response that includes expression of surface markers on DCs and secretion of inflammatory cytokines [20, 38]. We examined the expression of the co-stimulatory molecules CD40, CD80, and CD86 as well as MHC I and MHC II as evidence of DC activation. Both rCsHSP70 and rCsHSP90 stimulated surface molecule expression and cytokine secretion (i.e., IL-1β and -6 and TNF-α) in mBMDCs in a dose-dependent manner. High concentrations of rCsHSPs (≥ 5 μg/ml CsHSP90 and ≥ 25 μg/ml CsHSP70) also induced the secretion of IL-12 and -10 relative to PBS-treated control groups. In addition, mBMDCs acquired the capacity for production of intracellular IL-12 but not IL-10 in the presence of rCsHSPs. These results suggest that IL-10 is secreted early in the immune response. It is assumed that the capacity of DCs to capture and present antigens is diminished after initial activation [39]. We confirmed that both rCsHSP70 and rCsHSP90 stimulate cellular uptake and antigen presentation to mBMDCs. These results extend other reports that HSPs are potent activators of the innate immune system that can induce co-stimulatory molecule expression and pro-inflammatory cytokine secretion in mBMDCs [19, 20].

To evaluate T cell activation by rCsHSPs, mBMDCs activated with rCsHSPs were co-cultured with allogenic T cells. The number of T cells activated with rCsHSPs increased over time, and secretion of IL-2 and IFN-γ but not IL-4 was also increased in T cells obtained from allogenic as compared to PBS-treated control cells. IFN-γ was also secreted from splenocytes of rCsHSPs-immunized mice. These results demonstrate that rCsHSP70 and rCsHSP90 mainly induce the Th1 immune response involving CD8^+^ T cells or macrophages, which could potentially disable parasites.

HSPs conjugated with peptide have been shown to induce CD8^+^CTL responses [40, 41]. It has been reported that bacterial peptides elicit greater immune responses when bound to HSP [42]. In case of HSP from plants, HSP/antigen complexes have been shown to increase production of antibodies against a conjugated reporter antigen [43]. The Proline-rich (ProR) antigen of *C. sinensis* has been identified as a potential antigen [44]. In previous study, we identified that ProR peptide containing repeated sequences which derived from ProR antigen has strong antigenicity for clonorchiasis patients’ sera (data not shown). To ascertain the activity of CsHSP70 and CsHSP90 as an adjuvant for antigenic peptide, we designed the complex of CsHSPs bound with ProR peptides. Here we found that ProR peptide alone did not induce activation of mBMDCs. We speculated that rCsHSP/ProR peptide complexes would effectively induce an immune response, and generated CsHSP/ProR peptide complexes using ADP which increases peptide binding to HSP [18]. Incubation with CsHSP/ProR peptide complexes stimulated the secretion of TNF-α, IL-6, and especially IL-1β by mBMDCs but had no effect on surface marker expression. We also immunized C57BL/6 mice with CsHSP/ProR peptide complexes and measured antibody production and T cell proliferation. The peptide alone or mixed with commercial adjuvant did not induce a peptide-specific antibody response in mice, consistent with another report [45]. However, anti-ProR peptide antibody production was increased upon immunization with rCsHSP/ProR. Although rCsHSP90 more strongly stimulated mBMDCs and T cells in vitro, the in vivo antibody response was greater against rCsHSP70-bound peptide. On the other hand, T cell proliferation was not induced by CsHSP/peptide complex immunization. Nonetheless, our findings suggest that CsHSPs have an adjuvant effect.

## Conclusions

In conclusion, we confirmed that rCsHSP70 and rCsHSP90 are antigenic vaccine candidates since they activated the host immune response through cellular uptake and induction of antigen presentation. We also showed that CsHSPs can induce an in vitro immune response to ProR peptide derived from *C. sinensis* and have an adjuvant effect that includes the production of antibodies against ProR peptide. Our findings suggest that immunization with CsHSP-peptide conjugates can be an effective strategy for preventing *C. sinensis* infection or maturation.

## Abbreviations

APC: Antigen-presenting cell
APC: Allophycocyanin
CD: Cluster of differentiation
CFSE: Carboxyfluorescein succinimidyl ester
CTL: Cytotoxic T lymphocyte
DPBS: Dulbecco’s phosphate buffered saline
ELISA: Enzyme-linked immunosorbent assay
FA: Freund’s adjuvant
FBS: Fetal bovine serum
FITC: Fluorescein isothiocyanate
GM-CSF: Granulocyte-macrophage colony-stimulating factor
HSP: Heat shock protein
IFN: Interferon
IL: Interleukin
mBMDC: mouse bone marrow dendritic cell
MHC: Major histocompatibility complex
PE: Phycoerythrin
TNF: Tumor necrosis factor
